# Voluntary exercise could reduce sperm malformations by improving hypothalamus-hypophysis-gonadal axis and kisspeptin/leptin signaling in type 2 diabetic rats

**DOI:** 10.22038/IJBMS.2021.58740.13048

**Published:** 2021-12

**Authors:** Uldouz Kharazi, Rana Keyhanmanesh, Gholam Reza Hamidian, Saber Ghaderpour, Rafighe Ghiasi

**Affiliations:** 1 Student Research Committee, Tabriz University of Medical Sciences, Tabriz, Iran; 2 Department of Physiology, Faculty of Medicine, Tabriz University of Medical Sciences, Tabriz, Iran; 3 Drug Applied Research Center, Tabriz University of Medical Sciences, Tabriz, Iran; 4 Medical Education Research Center, Tabriz University of Medical Sciences, Tabriz, Iran; 5 Department of Basic Sciences, Faculty of Veterinary Medicine, University of Tabriz, Tabriz, Iran; 6 Stem Cell Research Center, Tabriz University of Medical Sciences, Tabriz, Iran

**Keywords:** Diabetes, Hypophysis, Hypothalamus, Kisspeptin, Leptin, Sperm malformations, Voluntary exercise

## Abstract

**Objective(s):**

Most male patients with type 2 diabetes mellitus (T2DM) experience infertility. It is well established that regular physical activity could alleviate diabetic infertility symptoms. This study was designed to determine the effect of voluntary exercise on sperm malformation.

**Materials and Methods:**

Thirty-two male Wistar rats were randomly divided into control (C), diabetic (D), voluntary exercise (Ex), and diabetic-voluntary exercise (D-Ex) groups. Diabetes was induced by an intraperitoneal injection of streptozotocin (35 mg/kg) followed by a high-fat diet for four weeks. Voluntary exercise was performed by placing the animals in the rotary wheel cages for ten weeks. Sperm malformations were analyzed. Moreover, the hypothalamic leptin, kisspeptin, kisspeptin receptors (KissR), as well as plasma LH, FSH, testosterone, and leptin levels were evaluated.

**Results:**

Results showed that induction of T2DM caused increased sperm malformation, plasma, and hypothalamic leptin as well as decreased hypothalamic kisspeptin, KissR, and plasma LH levels compared with the C group (*P*<0.001 to *P*<0.01). Voluntary exercise in the Ex group increased hypothalamic KissR, plasma FSH, LH, and testosterone levels compared with the C group; however, it decreased sperm malformation and hypothalamic leptin levels (*P*<0.001 to *P*<0.05). Voluntary exercise in the D-Ex group reduced sperm malformation, hypothalamic leptin, and plasma testosterone while elevated hypothalamic kisspeptin and KissR protein levels compared with the D group (*P*<0.001 to *P*<0.01).

**Conclusion:**

The results illustrated voluntary exercise reduces sperm malformations by improving the HHG axis and kisspeptin/leptin signaling in rats with T2DM.

## Introduction

The global prevalence of type two diabetes mellitus (T2DM) is increasing at an alarming rate. Based on the statistical data from the world health organization (WHO), the approximate number of T2DM cases was estimated at 177 million in 2000 and will rise to 300 million by 2025 (1). Evidence has shown that T2DM affects reproductive functions and leads to infertility. The increased incidence of infertility and the demand for treatment have become major issues worldwide (2-4). T2DM can affect male fertility directly or indirectly by impairing endocrine systems, leading to disrupted spermatogenesis (5). The male reproductive system is controlled by the hypothalamus-hypophysis-gonadal axis (HHG axis). This axis acts through gonadotropin-releasing hormone (GnRH) and two gonadotropin hormones: luteinizing hormone (LH) and follicle-stimulating hormone (FSH), which act synergistically to regulate spermatogenesis. T2DM can impair the HHG axis, decrease plasma levels of LH and FSH and ultimately, disrupt the process of androgen synthesis (6). 

The key regulator of the HHG axis is a specific peptide called kisspeptin. This is because kisspeptin could stimulate the hypothalamic GnRH release (7). 

Evidence shows that kisspeptin and its receptors (KissR) have a vital role in reproduction (8). In patients with T2DM, the plasma levels of kisspeptin are severely reduced. This could reduce reproductive hormones, including testosterone. Thus, T2DM could lead to hypogonadotropic hypogonadism (9). 

Reproductive activities demand a very high expenditure of energy. An adipocyte-derived cytokine called leptin is responsible for providing sufficient energy for these activities (10). Ample evidence is showing that leptin has a potential role in regulating reproductive functions. For example, leptin could regulate HHG-axis by controlling kisspeptin secretion (11). 

Previous investigations revealed that voluntary exercise could reduce diabetic-induced male reproductive complications (12, 13). However, its possible mechanisms have not been studied completely. Therefore, we aimed to find out the effect of voluntary exercise on sperm deformity index and malformation as well as the hypothalamic protein levels of kisspeptin, KissR, leptin, and plasma levels of leptin and gonadotropins. 

## Materials and Methods

***Experimental animals and study design***

In this experiment, thirty-two male Wistar rats (180–220 g and 6–7 weeks) were obtained from the laboratory animal house of Tabriz University of Medical Sciences. The animals were housed under controlled conditions (21–23 °C, 12 hr light-dark cycle, relative humidity 45–55%). All animals were provided free access to food and water for fifteen weeks. All cages were cleaned and the tape water was refilled every day. After the animals were acclimatized for one week, they were randomly allocated into the four following groups (n=8): control group (C), voluntary exercise group (Ex), diabetic group (D), and diabetic-exercise group (D-Ex). Animal care was in accordance with the Guide for the Care and Use of Laboratory Animals (NIH publication No.85-23, revised 1996) and approved by the Animal care Committee of Tabriz Medical Sciences University (approval number IR.TBZMED.REC.1398.308).

***Induction of type 2 diabetes***

Type 2 diabetes was induced by an intraperitoneal injection (IP) of streptozotocin (STZ) (35 mg/kg) followed by a high-fat diet (HFD) regimen, containing 48% carbohydrates, 20% protein, and 22% fat for 4 weeks (12-14). 

***Voluntary exercise protocol***

The rats in the Ex and the D-Ex groups were kept in separate cages with free-spinning wheels. The running distance of each was monitored daily for the following ten weeks with a digital rotation counter plugged into the rotary wheels. The total running distance was estimated by multiplication of the number of wheel rotations per day by wheel circumference. The cages with a wheel rotation of fewer than 2000 meters per day were excluded from the experiment (15). Daily running distances of rats in the D-Ex group were measured for ten weeks after confirmation of diabetes. 

***Blood sampling and tissue processing***

At the end of the experiment, all animals were anesthetized with an IP injection of 50 mg/kg ketamine and 5 mg/kg xylazine. The blood samples were collected from the inferior vena cava, centrifuged at 3500 revolutions per minute (RPM) for 10 min and then the samples were stored at -80 °C. 

The brains were dissected out from the skull and each hypothalamus was isolated and frozen in liquid nitrogen instantly and stored at -80 °C. The hypothalamus was isolated with a method formerly described by Saleh* et al.* (16). Briefly, the hypothalamus was isolated with an anterior coronal cut, which was anterior to the optic chiasma. Subsequently, a posterior coronal cut was applied at the posterior border of the mammillary bodies. 

To isolate the hypophysis from the thalamus, we used a method already described in 2017 (17). First, we lifted the hindbrain gently from the base of the skull with small forceps. We continued lifting the brain to remove the whole brain. Finally, the hypophysis was fully exposed. 

In the end, all animals were euthanized and sacrificed by decapitation. 

***Sperm morphology and sperm deformity index assessment***

The left epididymis was cut into small slices in 1 ml of Ham’s F10 medium (37 °C, 5% CO_2_ for 3–5 min) to make the spermatozoa swim out into the medium. Sperm malformation and sperm deformity index were analyzed in 500 extracted spermatozoa per animal in eosin–nigrosin stained smears (18). Sperm morphology assessment was performed following the WHO criteria using a bright-field microscope at the final magnification of 1000x. The sperm deformity index is a potent score for predicting male fertility (19). Sperm deformity index was calculated as a percentage as follows: the total number of the sperms with abnormal morphology was divided by the number of sperm randomly selected, irrespective of their morphological normality (19, 20). 

***Hormonal assay***

Commercially available rat enzyme-linked immunosorbent assay (ELISA) kits (Bioassay Technology Laboratory, Shanghai Korain Biotech Co. China) were used to measure the plasma levels of FSH, LH, testosterone, and leptin following the instructions of the manufacturers (21). 

***Western blotting***

The hypothalamic protein levels of kisspeptin, KissR, and leptin were measured with Western blot using a method previously described (22). Briefly, hypothalamus tissue homogenization was performed in Radio Immuno Precipitation Assay (RIPA) buffer supplemented with a mixture of protease inhibitors. The protein samples were centrifuged (Eppendorf Centrifuge 5415 R) at 12,000 g, 4 °C for 10 min, collected, and stored at -20 °C until processed. The protein samples were separated using 10% sodium dodecyl sulfate-polyacrylamide gel electrophoresis (SDS-PAGE). The protein bands were subsequently transformed onto a Polyvinylidene fluoride (PVDF) membrane (Roche, UK). Blots were blocked with 2 % milk in Tris-buffered saline solution (TBS-T). PVDF membranes were then incubated with primary antibodies β-actin (sc-47778, 1: 300) for 16–18 hr. PVDF membranes were washed 3 times for 15 min in a TBS-T buffer. The membranes were subsequently incubated with anti-rabbit secondary antibody (1:1000) for 75 min at room temperature. All antibodies were purchased from Santa Cruz (USA). Finally, membranes were placed in ECL prime Western blotting detection reagent (Amersham, UK), and the signals were visualized by exposure to autoradiography film (Kodak, USA). The density of the protein bands was quantitated using Image J 1.62 software (National Institutes of Health, USA). The membranes were normalized using an anti-β-actin (1:500, sc-130656) antibody as the marker. Luminescence and radiographic imaging were performed to visualize the protein bands. The sensitivity of the assay was pg/mm.

***Statistical analysis***

All data were analyzed with SPSS version 18. The analysis among the four groups and all parameters were analyzed by one-way ANOVA with Turkey’s *post-hoc* test. All data are presented as the mean ± SEM. A probability value of <0.05 was considered statistically significant. 

## Results

***Effect of voluntary exercise on the sperm deformity index***

**Figure 1:**
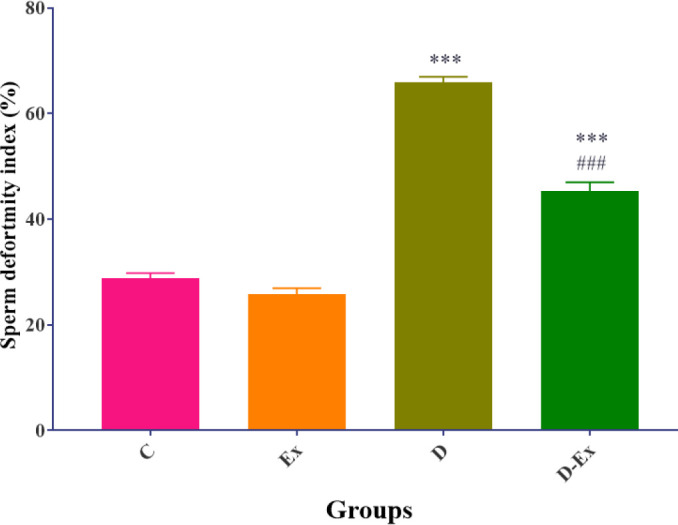
Effect of voluntary exercise on the sperm deformity index in the control group (C), type 2 diabetic group (D), voluntary exercise group (Ex), and type 2 diabetic-voluntary exercise group (D-Ex) (all groups, n=6). Data are expressed as mean ± SEM. ***; *P*<0.001 shows the statistical difference between C and different groups. ###; *P*<0.001 shows the statistical difference between D and the D-Ex groups

The results showed that the sperm deformity index was significantly increased in the D and the D-Ex groups compared with the C group (*P*<0.001). Voluntary exercise significantly improved the sperm deformity index in the D-Ex group in comparison with the D group (*P*<0.001, Figure 1). 

***Effect of voluntary exercise on the sperm malformation***

**Figure 2:**
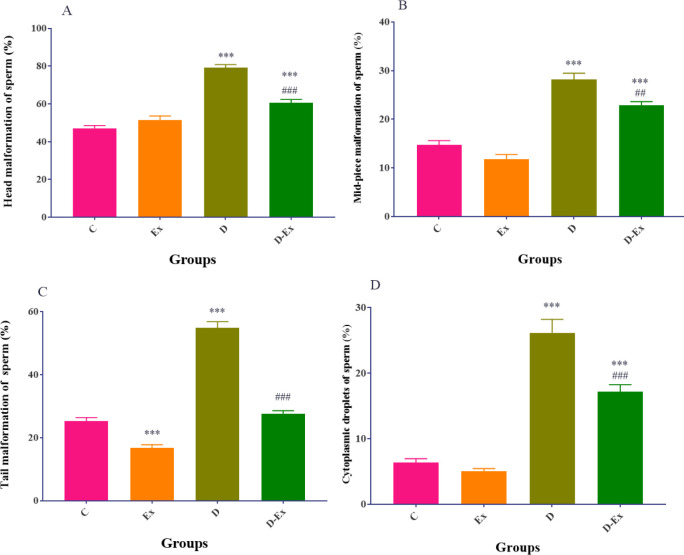
The effect of voluntary exercise on malformations of the head (A), mid-piece (B), tail (C), and cytoplasmic droplets (D) of sperm in the control group (C), type 2 diabetic group (D), voluntary exercise group (Ex), and type 2 diabetic-voluntary exercise group (D-Ex) (all groups, n=6). Data are expressed as mean ± SEM. ***; *P*<0.001 shows the statistical difference between C and different groups. ###; *P*<0.001 shows the statistical difference between D and the D-Ex groups

Our findings revealed that the malformations of the head, mid-piece, tail, and cytoplasmic droplets of sperm in the D and D-Ex groups were significantly elevated compared with the C group (*P*<0.001). Voluntary exercise remarkably improved these malformations in the D-Ex group in comparison with the D group (*P*<0.001, Figure 2A-D). 

***Effect of voluntary exercise on hypothalamic protein levels of kisspeptin and KissR***

**Figure 3:**
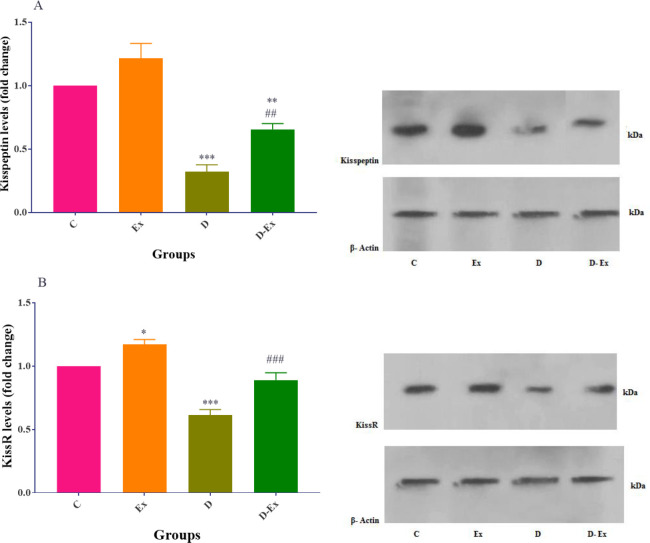
Effect of voluntary exercise on the hypothalamic levels of kisspeptin (A) and KissR (B) in the control group (C), type 2 diabetic group (D), voluntary exercise group (Ex), and type 2 diabetic-voluntary exercise group (D-Ex) (all groups, n=8). Data are expressed as mean ± SEM. *; *P*<0.05, **;* P*<0.01, and ***; *P*<0.001 show the statistical difference between C and different groups. ##; *P*<0.01, and ###; *P*<0.001 show statistical difference between D and the D-Ex groups

The results indicated that there was a statistical difference between the hypothalamic protein levels of kisspeptin in D and D-Ex groups compared with the C group (*P*<0.001 to *P*<0.01). Voluntary exercise non-significantly increased this parameter in the Ex group compared with the C group. Voluntary exercise significantly increased this parameter in the D-Ex group in comparison with the D group (*P*<0.01, Figure 3A).

In the D and the D-Ex groups, KissR protein levels in the hypothalamus of rats were remarkably reduced in comparison with the C group (*P*<0.001). This parameter was significantly elevated in the Ex group compared with the C group (*P*<0.05). Voluntary exercise significantly enhanced this parameter in the D-Ex group compared with the D group (*P*<0.001, Figure 3B).

***Effect of voluntary exercise on hypothalamic and plasma levels of leptin***

**Figure 4:**
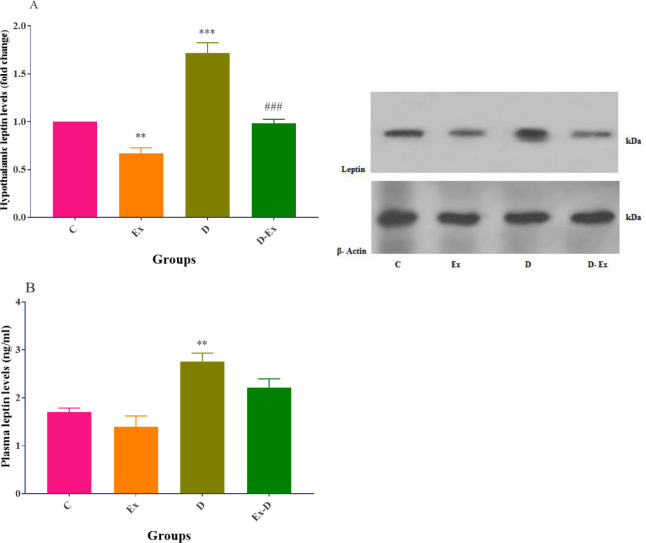
Effect of voluntary exercise on hypothalamic (A) and plasma (B) levels of leptin in the control group (C), type 2 diabetic group (D), voluntary exercise group (Ex), and type 2 diabetic-voluntary exercise group (D-Ex) (all groups, n=8). **; *P*<0.01 and ***; *P*<0.001 show the statistical difference between C and different groups. ###; *P*<0.001 shows the statistical difference between D and the D-Ex groups

The findings indicated that the hypothalamic leptin levels were elevated in the D group compared with the control group (*P*<0.001). Voluntary exercise significantly decreased this parameter in the Ex group compared with the C group (*P*<0.01). Voluntary exercise led to a reduction in this parameter in the D-Ex group in comparison with the D group (*P*<0.001, Figure 4A).

The plasma levels of leptin in the D group were notably increased compared with the C group (*P*<0.01), but voluntary exercise non-significantly decreased this parameter compared with the C group. 

In the D-Ex group, this parameter was non-significantly reduced in comparison with the D group (Figure 4B).

***Effect of voluntary exercise on plasma levels of FSH, LH, and testosterone***

**Figure 5:**
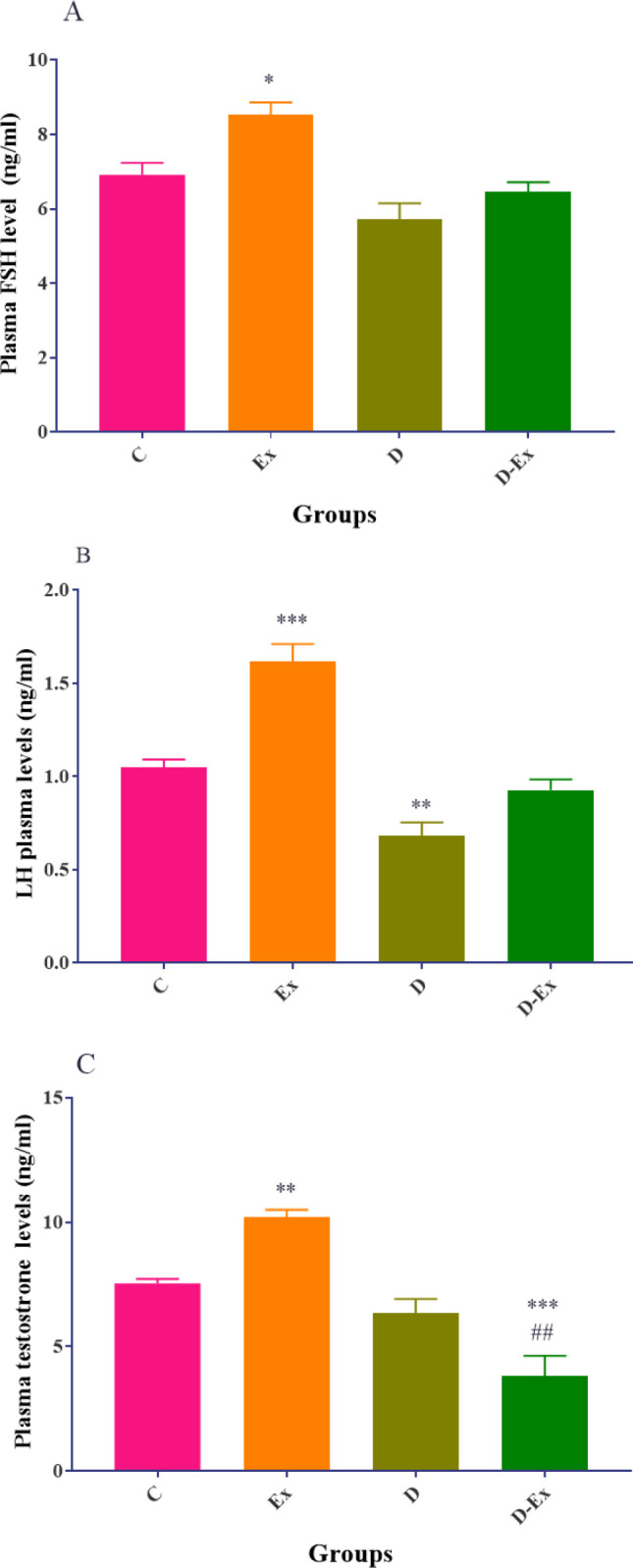
Effect of voluntary exercise on the plasma levels of FSH (A), LH (B), and testosterone (C) in the control group (C), type 2 diabetic group (D), voluntary exercise group (Ex), and type 2 diabetic-voluntary exercise group (D-Ex) (all groups, n= 6). *; *P*<0.05, **; *P*<0.01, and ***; *P*<0.001 show the statistical difference between C and different groups. ##; *P*<0.01, and ###; *P*<0.001 show statistical difference between D the D-Ex groups

There was a significant reduction in the plasma levels of LH in the D group compared with the C group (*P*<0.01), whereas the plasma levels of FSH and testosterone were non-significantly decreased in the D group than the C group. The plasma levels of FSH, LH, and testosterone in the Ex group were increased significantly compared with the C group (*P*<0.001 to *P*<0.05). Voluntary exercise decreased the plasma levels of testosterone in the D-Ex group compared with the D group (*P*<0.01). However, the plasma levels of FSH and LH were non-significantly increased in the D-Ex group compared with the D group (Figure 5A-C). 

## Discussion

In this study, we analyzed the effect of voluntary exercise on sperm deformity index and sperm malformation, plasma levels of reproductive hormones, hypothalamic protein levels of leptin, kisspeptin, and KissR. 

The findings of the present study showed that induction of T2DM increased the sperm deformity index and deteriorated sperm morphology. Among sperm parameters, sperm morphology is one of the most important semen analyses that is accepted as a good indicator of infertility and sub-fertility (23). The population of sperms with abnormal morphology is high in patients with T2DM (24). One of the underlying reasons is the increased levels of reactive oxygen species (ROS) (25-27). ROS are highly active molecules that target cellular lipid and protein contents, leading to a loss of sperm plasma membrane integrity (28). These data are consistent with our previous studies, which revealed that sperm morphology was impaired in type 1 diabetic male rats (29-31). Moreover, other studies have shown that sperm morphology is severely impaired in patients with T2DM (32, 33). 

The results represented that voluntary exercise for ten weeks improved sperm deformity index and sperm malformations. The results of this study agree with the previous studies. A study in 2016 showed that resistance exercise training for five days a week for the following ten weeks could not improve sperm morphology in male rats with T2DM**.** This could be explained by a decrease in testosterone levels (34). Another study in 2011 analyzed the effect of high altitudes at 5,900 meters on sperm parameters in the experienced male mountaineers (35). Semen analysis showed that physical training did not affect sperm morphology and viability. The results concluded that sperm differentiation during meiosis was adapted to prolonged physical training at the high altitude. Moreover, Vaamonde *et al.* in 2011 showed that sperm morphology was improved in the exercising group that was subjected to regular endurance exercising for more than a year (36). The explanation for these results was the elevated FSH and LH levels, which could improve spermatogenesis and increase testosterone levels respectively.

The findings of our study revealed that induction of T2DM diminished the hypothalamic protein levels of kisspeptin and KissR. Kisspeptin; a peptide with 54 amino acids, is considered a regulatory key in sexual maturity. There is a strong correlation between kisspeptin and reproductive hormones. A human study in 2005 showed that LH, FSH, and testosterone levels were elevated in response to kisspeptin 54 administration (37). Moreover, Navarro *et al.* in 2004 showed that ICV administration of kisspeptin could significantly elevate LH levels and hypothalamic kisspeptin and KissR mRNA expression at the pre-pubertal or adulthood stage in both male and female rats (38). 

The findings of the present study were consistent with previous studies. One study in 2019 demonstrated that plasma levels of kisspeptin were severely reduced in T2DM male patients (8.34 ng/ml) compared with the healthy control group (16.26 ng/ml). The measurements showed that FSH and LH levels were higher in T2DM patients compared with the healthy control group. This could be explained by the decreased testosterone levels, which failed to exert negative feedback on the HHG axis (39). 

In the present study, voluntary exercise could elevate the hypothalamic protein levels of kisspeptin and KissR in diabetic conditions. In one study, *Arisha et al.* in 2019 showed that 60 min of chronic swimming exercise per day, five days a week for the following six weeks could significantly decrease testicular KissR mRNA expression, hypothalamic kisspeptin mRNA expression, and their protein levels as well (40). This study also showed that sperm quality, number, viability, and motility were markedly reduced. The reason was that chronic exercise could severely disrupt reproductive functions by increasing oxidative stress in the testicular and the hypothalamic tissues (41-44). One of the reasons that testis are vulnerable to ROS is an abundance of polyunsaturated fatty acids present in the cell membrane, leading to a deterioration of testosterone synthesis processes (41). ROS can also attack GnRH neurons and ultimately, cause infertility (45). 

Our findings showed that T2DM increased the hypothalamic and plasma levels of leptin. Kisspeptin and leptin can together regulate reproductive functions. However, leptin resistance could contribute to infertility by counteracting the kisspeptin/KissR system activity in patients with T2DM (46). The results of this study agree with the previous studies. A human study in 2003 revealed that leptin levels were potentially related to insulin resistance and beta-cell function in diabetic patients (47).

The results indicated that voluntary exercise for 10 weeks decreased the hypothalamic and plasma levels of leptin. These results were consistent with previous studies. In one study, Haskell-Luevano *et al*. in 2009 showed that eight weeks of voluntary exercise could decrease plasma leptin levels up to 77% in obese melanocortin-4 receptor knockout mice (48). Another study in 2007 showed that in high-runner female rats, six days of voluntary exercise could decrease plasma leptin levels (1.5 to 3.5 ng/ml) compared with the non-exercising groups (49).

The results indicated that secretion of reproductive hormones especially LH was disturbed in diabetic animals. Studies have shown that inappropriately low LH, FSH, and testosterone levels in patients with T2DM could adversely affect spermatogenesis (50, 51). The findings of this study agree with the previous studies. Researchers in 2004 analyzed concentration of testosterone, LH, and sex hormone-binding globulin (SHBG) and the incidence of hypogonadotropic hypogonadism in diabetic subjects (52). They showed that decreased FSH and LH levels could decrease testosterone synthesis and induce hypogonadism. 

In the present study, we showed that ten weeks of voluntary exercise could significantly increase the plasma levels of FSH, LH, and testosterone in both diabetic and non-diabetic conditions. There are different reports about the alterations of reproductive hormones during vigorous physical activity, indicating decreased testosterone and unchanged LH and FSH levels (53-55)

The results of our study are consistent with a previous study. It showed that chronic high-intensity exercise for 1, 2, or 3 hr per day, five days a week for the following four weeks could significantly decrease LH and testosterone levels in male rats. FSH levels did not show any significant changes. This could be explained by a decrease in the activity of steroidogenic enzymes such as 3β-hydroxysteroid dehydrogenase (3β-HSD) and 17β-hydroxysteroid dehydrogenase (17β-HSD). This study also, showed that chronic high-intensity exercise could disrupt spermatogenesis by inducing oxidative stress. This could be explained by the fact that the activity of the antioxidant enzymes was severely impaired. These enzymes included superoxide dismutase (SOD), catalase (CAT), peroxidase, and glutathione-s-transferase (GST). These enzymes play important roles in reducing free radicals. For example, GST plays an important role in reducing hydrogen peroxide levels and organic peroxides (56, 57). CAT could interact with 17β-HSD. Moreover, there is a positive correlation between 17β-HSD and peroxidase (58). Therefore, CAT and peroxidase could play a role in testosterone biosynthesis. 

Since voluntary exercise could improve sperm malformation, it can be considered a therapeutic option for preventing the deleterious effects of diabetes on the reproductive activity of males. The first mechanism of action lies in the enhanced levels of reproductive hormones. This could be explained by the increased kisspeptin and KissR levels. The Kisspeptin system could enhance the activity of the HHG axis and ultimately reset LH, FSH, and testosterone levels back to normal (59). The second mechanism of action is that voluntary exercise could improve sperm index and malformation through decreasing tissue and plasma levels of leptin. Leptin could directly act on germ cells and induce oxidative stress and DNA damage. Therefore, leptin resistance could increase the population of abnormal sperm directly or indirectly (60).

## Conclusion

The results of the present study illustrated that voluntary exercise reduced sperm malformations through improving the HHG axis, kisspeptin/KissR system, and decreasing leptin levels in rats with T2DM. Voluntary exercise increased the plasma levels of FSH, LH, and testosterone and thus, alleviated the deteriorating effects of T2DM on sperm morphology. Moreover, voluntary exercise reduced the plasma and tissue levels of leptin, which contributed further to improving the HHG axis. Accordingly, the effectiveness of voluntary exercise should be considered in improving the reproductive health of male patients with T2DM.

## Authotrs’ Contributions

UK Data curation, formal analysis, writing the original draft; GRH Validation, visualization; SC Data curation; RK Supervision, validation, visualization, writing, review, and editing; RG Conceptualization, data curation, formal analysis, methodology, project administration, supervision, validation, visualization, writing the original draft, writing, review, and editing. 

## Funding

This manuscript is part of an MSc research project (Code: IR.TBZMED.REC.1397.303) and is supported by a grant from the Drug Applied Research Center, Tabriz University of Medical Sciences, Tabriz, Iran.

## Conflicts of Interest

The authors declare no conflicts of interest.
